# Preparation, Characterization, and Application of AlN/ScAlN Composite Thin Films

**DOI:** 10.3390/mi14030557

**Published:** 2023-02-27

**Authors:** Laixia Nian, Yuanhang Qu, Xiyu Gu, Tiancheng Luo, Ying Xie, Min Wei, Yao Cai, Yan Liu, Chengliang Sun

**Affiliations:** 1 The Institute of Technological Sciences, Wuhan University, Wuhan 430072, China; 2 School of Physics and Technology, Wuhan University, Wuhan 430072, China; 3 School of Microelectronics, Wuhan University, Wuhan 430072, China

**Keywords:** composite film, AlN/ScAlN, lamb wave resonator, first principal calculation

## Abstract

Piezoelectric aluminum nitride (AlN) thin film, as a commonly used material for high-frequency acoustic resonators, has been a research hotspot in the RF field. Doping Sc elements in AlN is one of most effective methods to improve the piezoelectricity of the material. In this work, the first principal calculation and Mori–Tanaka model are used to obtain the piezoelectric constants of AlN, ScAlN, and AlN/ScAlN composites. Then, five types of AlN/ScAlN thin films are prepared on 8 inch silicon substrates. The crystal quality, roughness, and stress distribution are measured to characterize the film quality. The results show that composite film can effectively solve the problem of abnormal grains and reduce the roughness. Finally, a lamb wave resonator with an AlN/Sc_0.2_Al_0.8_N composite working at 2.33 GHz is fabricated. The effective electromechanical coupling coefficient *K*_eff_^2^ is calculated to be 6.19%, which has the potential to design high-frequency broadband filters.

## 1. Introduction

The RF filter is one of the core components of wireless communication and is widely used in both the military and civilian fields. The RF resonator is the basic unit of the filter, and it is the core component that determines the performance of the filter [1]. Despite them being the current favorites in the field of RF filters, surface acoustic wave (SAW) and thin film bulk acoustic wave (FBAR) technologies still have their own shortcomings. Although SAW resonators have an easy fabrication process and a low cost, they cannot reach frequency bands above 3 GHz due to the limitations of lithography and the low acoustic velocity [2]. In addition, SAW also has disadvantages such as low power handling, a large temperature drift coefficient, and a low-quality factor (Q) [3]. FBAR devices based on aluminum nitride (AlN) have the advantages of a high frequency, a high power, a high Q, and a small temperature drift [4]. However, since its resonant frequency depends on the thickness of the piezoelectric materials and electrodes, it is difficult to realize the monolithic integration of multi-band filters. With the advent of the 5G era, the monolithic integration of small-volume multi-band RF filters has become the development trend of future RF front-ends. Lamb wave resonators (LWRs) based on AlN thin film materials have the advantages of multi-frequency coexistence within one chip [5,6].

The AlN film has the advantages of a high acoustic velocity, a high thermal conductivity, and a good thermal stability, but its piezoelectric coefficient (d_33_ = 6.5 pC/N) and electromechanical coupling coefficient *K*_t_^2^ are low, which is not conducive to realizing the filter with a large bandwidth and low loss [7,8,9,10]. Doping elements, such as Sc, can significantly increase the piezoelectric coefficient d_33_ of the AlN film, thereby obtaining a larger electromechanical coupling coefficient *K*_t_^2^ [11,12]. The ScAlN material is used to replace the AlN piezoelectric film to prepare high-frequency, large-bandwidth LWRs. However, pure ScAlN films have many defects, such as massive abnormal grains, poor temperature sensitivity, and high dielectric loss [13,14,15,16]. Minghua Li et al. found that when physical vapor deposition (PVD) was used to prepare ScAlN thin films, large-sized grains appeared on the surface of the thin films [17].

In this work, AlN/ScAlN composite films are prepared to solve the above problems. The AlN thin film can be used as a compensation layer to balance the advantages and disadvantages of pure ScAlN. Compared with pure ScAlN, the crystal orientation, surface roughness, and film stress of composite films are all improved. The first principal calculation is used to calculate the piezoelectric constants of the different piezoelectric films. The piezoelectric constants of the composite films are then deduced using the Mori–Tanaka (MT) model. Finally, LWRs devices based on AlN/ScAlN thin films are fabricated and tested. 

## 2. Modelling and Calculation

**Figure 1:**
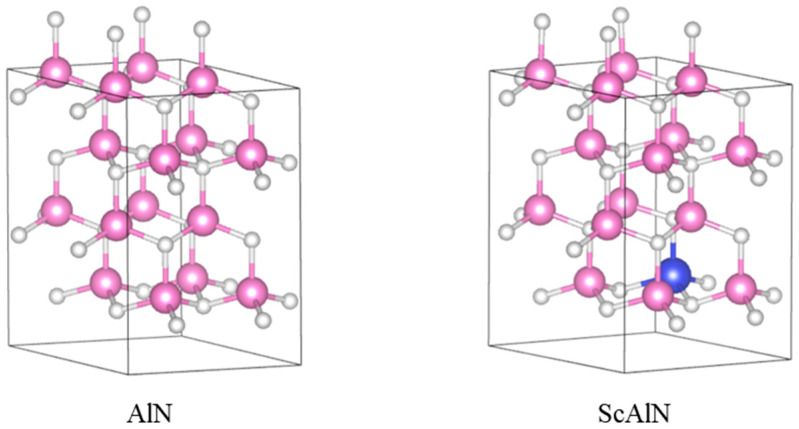
The wurtzite structure of AlN and ScAlN.

**Figure 2:**
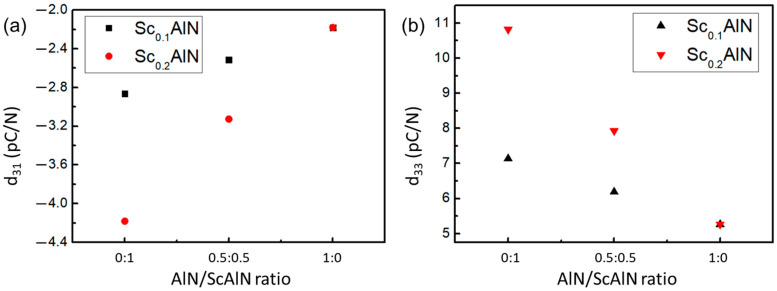
The calculated piezoelectric constant (**a**) d_31_ and (**b**) d_33_ of AlN and AlN/ScAlN composite films. The value of pure AlN and ScAlN are obtained by first principal calculation and the value of composites are obtained by MT model.

Figure 1 shows the crystalline composition of AlN and ScAlN supercell in this work. The piezoelectric properties of AlN and ScAlN materials are derived from first-principle calculations using Materials Studio (MS) and Vienna Ab-initio Simulation Package (VASP) software. The lattice constant a, b, and c of pure AlN are 0.311 nm, 0.311 nm, and 0.498 nm, respectively. Replacing Al atoms with Sc atoms in each supercell model enables variations in the doping content. The generalized gradient approximation of Perdew–Burke–Ernzerhof (GGA-PBE) is used, and the plane wave cutoff is set to 600 eV. Figure 2 shows the calculation results. With the introduction of the Sc element in AlN, the piezoelectric strain constants d_31_ and d_33_ both increase. The d_31_ (−4.18 pC/N) and d_33_ (10.818 pC/N) of Sc_0.2_Al_0.8_N are almost twice that of pure AlN (−2.181 pC/N and 5.268 pC/N, respectively). The piezoelectric coefficients d_31_ and d_33_ of Sc_0.1_Al_0.9_N increase a little, reaching −2.86 pC/N and 7.14 pC/N, respectively.

In order to calculate the piezoelectric constant of the AlN/ScAlN composite film. The Reuss model “iso-stress” [18] is used. Additionally, the electro-elastic modulus matrix Σ of AlN/ScAlN composite films can be described as:

(1) $$\Sigma ={\left[\begin{array}{cc} c & e^{T}\\ e & -\varepsilon \end{array}\right]}^{-1}$$

where c is the fourth-order elastic stiffness matrix, *e* is the piezoelectric modulus tensor, and ε is the dielectric modulus tensor. 

The MT mode is one of the effective methods used to calculate and predict the effective piezoelectric constants of composite films [19]. For AlN/ScAlN composite films, the effective modulus Σ* modulus can be described as [20]:

(2) $$\Sigma^{*}=\Sigma_{1}+v_{2}\left(\Sigma_{2}-\Sigma_{1}\right)B^{MT}$$

where Σ_1_ and Σ_2_ present the electro-elastic modulus of AlN and ScAlN, respectively, and *v*_2_ is the volume faction of the ScAlN phase. *B^MT^* is the volume-dependent modulus which can be calculated by the ‘dilute’ approximation, as follows:

(3) $$B^{MT}=B^{dil}{\left[\left(1-v_{2}\right)I+v_{2}B^{dil}\right]}^{-1}$$

Then, the concentration tensor $B^{dil}\;$ can be written as:

(4) $$B^{dil}={\left[I+A\sum_{1}^{-1}\left(\Sigma_{2}-\Sigma_{1}\right)\right]}^{-1}$$

where *I* is the 9 × 9 identity matrix, and *A* is the Eshelby tensor. According to the properties of the electroelastic modulus [19] and Equation (2), the effective piezoelectric-strain constants of AlN/ScAlN composite films are obtained.

(5) $$d_{31}^{*}=-\frac{\Sigma_{91}^{*}}{\Sigma_{99}^{*}}$$

(6) $$d_{33}^{*}=-\frac{\Sigma_{93}^{*}}{\Sigma_{99}^{*}}$$

## 3. Preparation and Characterization

In order to determine the structural and crystal quality, 1μm thick pure AlN, pure ScAlN, and the AlN/ScAlN composite film were prepared on 8 inch low-resistivity (1–5 Ωcm) Si (100) wafers by using a sputtering system (SPTS Sigma). The non-contact thin film metrology system (NanoSpec 9300) is used to measure the thickness of pure AlN and ScAlN films. Because the equipment cannot measure the composite film, the thickness of the composite film is deposited according to the deposition rate of the pure film. The AlN target utilized is 12 inch target with 99.999% purity. The Sc_0.1_Al_0.9_N and Sc_0.2_Al_0.8_N alloy targets utilized are 12 inch targets with 99.95% purity. Before deposition, the wafers are pre-warmed and sputtering etched to remove the surface oxidation layer. Four dummy wafers were processed at the beginning for thermalizing the chamber to the process temperature, and also, for cleaning of the chamber and target. For this work, reactive sputter deposition film control techniques included the variation in the RF power, platen temperature, and gas flow ratios between Ar and N_2_. During growth, a heater temperature of 200 °C was used. The detailed preparation parameters are listed in Table 1, and these have been optimized to obtain high-quality films. 

**Figure 3:**
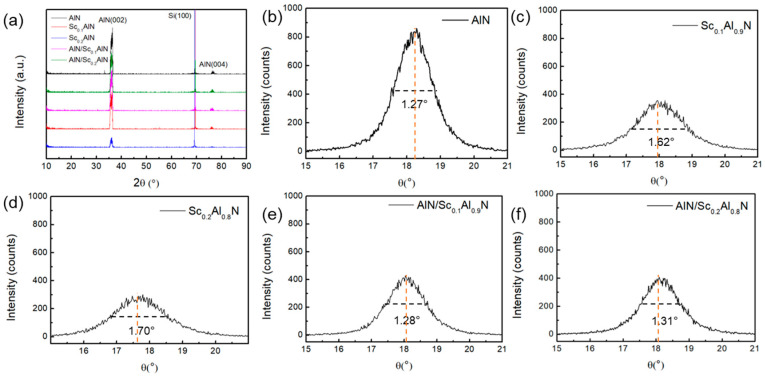
The XRD of AlN, ScAlN, and AlN/ScAlN composite films: (**a**) the θ-2θ pattern of five types of films and FWHM of (**b**) AlN, (**c**) Sc_0.1_Al_0.9_N, (**d**) Sc_0.2_Al_0.8_N, (**e**) AlN/Sc_0.1_Al_0.9_N, (**f**)AlN/ Sc_0.2_Al_0.8_N.

Rocking curve measurements (PANalytical X’Pert Pro) are taken for the analysis of the orientation and the crystalline quality of the films, as shown in Figure 3. Using AlN as a compensation layer improves the FWHM to 1.28°/1.31° compared to those of the pure Sc_0.1_Al_0.9_N (1.62°) and Sc_0.2_Al_0.8_N (1.70°). This is further strengthened by the results of the composite tests. In the measurement, (002) columnar wurtzite growth is observed at around 18°. With the increase in the Sc element concentration, (002) reflection happens to shift to a lower diffraction angle. The phenomenon is due to the changed *c*-lattice constant of ScAlN compared to that of AlN. According the XRD data, the c-lattice constants are calculated as 4.919, 5.012, 5.062, 4.945, 4.958 for AlN, Sc_0.1_Al_0.9_N, Sc_0.2_Al_0.8_N, AlN/Sc_0.1_Al_0.9_N, and AlN/Sc_0.2_Al_0.8_N, respectively. 

**Figure 4:**
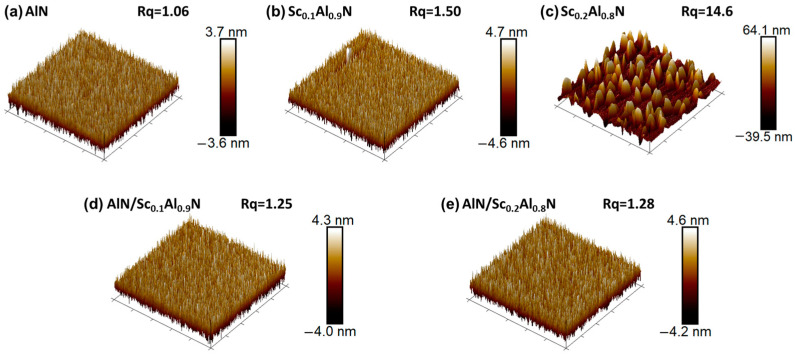
The AFM of (**a**) AlN, (**b**) Sc_0.1_Al_0.9_N, (**c**) Sc_0.2_Al_0.8_N, (**d**) AlN/Sc_0.1_Al_0.9_N, and (**e**) AlN/Sc_0.2_Al_0.8_N films.

Furthermore, an Atomic Force Microscope (AFM, Bruker Dimension^®^ Icon™) is used to observe the surface roughness and condition, as shown in Figure 4. Typically, the material in the center of the wafer exhibits larger defects, so a 5 × 5 μm area in the center of the wafer is used to check for abnormal grains. These abnormal grains have a conical shape, widening along the growth direction. Abnormal grains nucleate during growth, randomizing the film texture and deteriorating the desired piezoelectric properties of the film. For pure piezoelectric films, Sc_0.2_Al_0.8_N exhibits more abnormal grains, while Sc_0.1_Al_0.9_N exhibits fewer abnormal grains. Composite films effectively solve this issue. From the measured results, the roughness values of the AlN/Sc_0.1_Al_0.9_N and AlN/Sc_0.2_Al_0.8_N composite films are 1.25 and 1.28, respectively, while those of pure ScAlN are 1.50 and 14.6.

**Figure 5:**
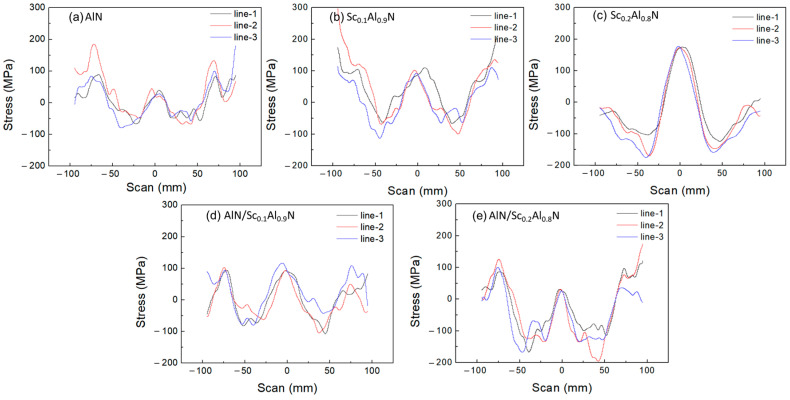
The stress distribution of (**a**) AlN, (**b**) Sc_0.1_Al_0.9_N, (**c**) Sc_0.2_Al_0.8_N, (**d**) AlN/Sc_0.1_Al_0.9_N, and (**e**) AlN/ Sc_0.2_Al_0.8_N films.

In addition, film stress is measured (FSM 128-NT) for five types of films. The three data lines correspond to three measurements, and each scan means that the wafer is rotated 120° for a new measurement. For comparison, the sputtering power for thin film deposition is adjusted to keep the average stress at almost zero. From the measurement results in Figure 5, it can be seen that pure Sc_0.2_Al_0.8_N has the largest stress range. The composite film also help to reduce the stress distribution range.

## 4. Fabrication and Experiments

**Figure 6:**
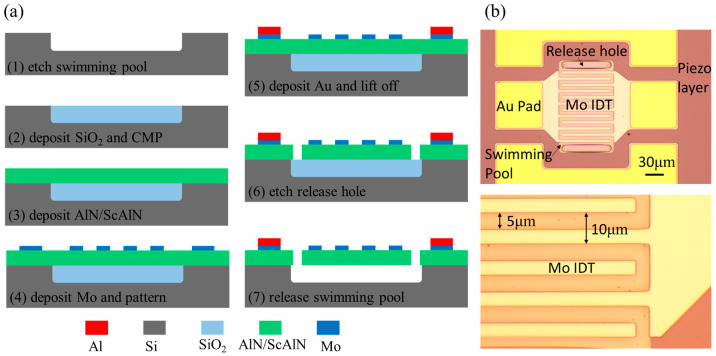
(**a**) The process flow of LWR with composite piezoelectric layers. (**b**) The optical image of fabricated LWR device.

Figure 6a show the detailed process of lamb wave resonating using AlN/ScAlN composite films. First, a 2.5 μm swimming pool is etched by Deep Reactive Ion Etching (DRIE). Then, SiO_2_ is deposited on the surface of the swimming pool. Mechanical chemical polishing is used to flatten the wafer. Next, 1 μm AlN/ScAlN composite piezoelectric films are deposited. The composite film must be kept in a vacuum when one is transferring the wafer from the AlN chamber to the ScAlN chamber. Two hundred nm top electrodes are deposited and patterned. Then, 1 μm Au pads are evaporated and lift-off. The release holes are patterned and etched. Finally, the BOE is used to release the swimming pool to form the air cavity. Figure 6b shows the optical images of the LWR device. The pitch of the LWR is 10 μm, and Inter Digital Transducers (IDTs) length is 80 μm. 

**Figure 7:**
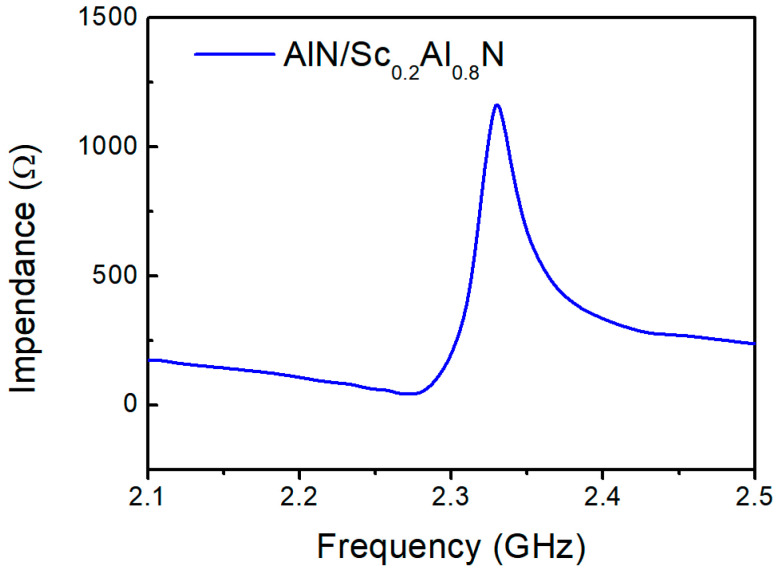
The impedance curve of fabricated lamb wave resonator based on AlN/Sc_0.2_Al_0.8_N composite film.

S-parameters of the resonators are measured using a network analyzer (Keysight N5222B) through two GSG probes, and then converted to the resonator’s impedance. The frequency range is from 2.1 GHz to 2.5 GHz, with a step of 1 MHz. Figure 7 shows the measured impedance curves of LWR based on AlN/Sc_0.2_Al_0.8_N films. The measured results show that the resonant frequency and anti-resonant frequency are 2.27 GHz and 2.33 GHz, respectively. The effective coefficient *K*_eff_^2^ are calculated as 6.2%. Compared with other works, it is found the LWR device with the AlN/ScAlN composite film have a large *K*_eff_^2^ value, which is nearly twice that of pure Sc_0.2_Al_0.8_N and is similar to that of pure Sc_0.4_Al_0.6_N, as shown in Table 2. 

## 5. Conclusions

In this work, AlN/ScAlN composite films are prepared, characterized, and applied. The first principal calculation and MT model are used to obtain the piezoelectric constants. The results show that the AlN/ScAlN composites have not only moderate piezoelectricity, but they also have fewer defects, less roughness, and a better film quality. Based on AlN/Sc_0.2_Al_0.8_N composite films, the LWR device is fabricated with good electrical and acoustic performances.

## Figures and Tables

**Table 1:** The main preparation parameter of AlN, Sc_0.1_Al_0.9_N, and Sc_0.2_Al_0.8_N thin film.

Material	AlN	Sc_0.1_Al_0.9_N	Sc_0.2_Al_0.8_N
Target power (kW)	6	10	10
Pulsing frequency (kHz)	100	100	100
Temperature (°C)	200	200	200
Ar flow (sccm)	25	23	19
N_2_ flow (sccm)	155	120	105
Base pressure (Torr)	<5 × 10^−8^	<9 × 10^−8^	<2 × 10^−7^

**Table 2:** Comparison of previous work with *K*_eff_^2^.

Material	*f*(GHz)	*K*_eff_^2^
AlN [21]	0.83	1.36
Sc_0.2_Al_0.8_N [22]	0.24	4.5
Sc_0.2_Al_0.8_N [22]	0.53	3.6
Sc_0.4_Al_0.6_N [23]	2.63	7.39
This work	2.33	6.19

## Data Availability

Data and code are available from the corresponding authors upon reasonable request.
